# SaMDE: A Self Adaptive Choice of DNDE and SPIDE Algorithms with MRLDE

**DOI:** 10.3390/biomimetics8060494

**Published:** 2023-10-18

**Authors:** Pravesh Kumar, Musrrat Ali

**Affiliations:** 1 REC Bijnor, Chandpur 246725, India; praveshtomariitr@gmail.com; 2 Department of Basic Sciences, PYD, King Faisal University, Al Ahsa 31982, Saudi Arabia

**Keywords:** differential evolution, optimization, evolutionary algorithms, mutation, self-adaptive

## Abstract

Differential evolution (DE) is a proficient optimizer and has been broadly implemented in real life applications of various fields. Several mutation based adaptive approaches have been suggested to improve the algorithm efficiency in recent years. In this paper, a novel self-adaptive method called SaMDE has been designed and implemented on the mutation-based modified DE variants such as modified randomized localization-based DE (MRLDE), donor mutation based DE (DNDE), and sequential parabolic interpolation based DE (SPIDE), which were proposed by the authors in previous research. Using the proposed adaptive technique, an appropriate mutation strategy from DNDE and SPIDE can be selected automatically for the MRLDE algorithm. The experimental results on 50 benchmark problems taken of various test suits and a real-world application of minimization of the potential molecular energy problem validate the superiority of SaMDE over other DE variations.

## 1. Introduction

Optimization problems occur almost in each field of engineering and science branches. In general, these problems may be classified as non-linear, non-convex non-continuous, non-differentiable, or having several local optimum values and therefore solving these problems is beyond the capacity of traditional methods due to their certain limitations. As a result, so many evolutionary algorithms (EAs) like Particle swarm optimization (PSO), Differential evolution (DE), Artificial Bee colony (ABC), Cuckoo Search (CS), Teaching Learning based optimization (TLBO), Gray Wolf optimization (GWO), Reptile Search Algorithm (RSA), Whale Optimization Algorithm (WOA), and Manta Ray Foraging Optimization (MRFO), etc., have emerged during some past years to handle such complicated situations. The prime benefit of EAs over the traditional system is that they only need the objective function, whereas the other calculus properties like differentiability and continuity are not necessarily important.

The Differential evolution algorithm (DE) [1] comes under the EAs categories and has gained a reputation since the last few years as a highly capable and robust optimizer. There are many reasons for its popularity among the researchers such as its compact size, requirements of only few control parameters, easy implementation quality, quick convergence rate, etc. Due to its many advantages, it has been frequently used to deal with large scale, constrained, dynamic, multi-objective and multimodal optimization problems.

Despite many positive attributes, DE also has some deficiencies like population diversity and stagnation problems and consequently it does not work in many situations or gives a slow convergence speed. Alot of research has been carried out in the past two decades to reduce its deficiencies and make it a more efficient algorithm.

Initially Storn & Price [1] and Liu & Lampinen [2] have suggested that control parameters *F* and *Cr* should have a value between (0.5, 1) and (0.8, 1), respectively. Later, several studies have been carried out on the selection of suitable parameter settings for DE. A decent literature survey for control parameters may be found in [3]. To keep away from the manual tuning of parameters, researchers have suggested adaptive/ self-adaptive setting of parameters, where the control parameters are changed vigorously based on the response of the search space in place of taking a fixed value. A few works in the improvement of adaptive/self-adaptive methods of control parameters values are suggested in [4,5,6,7,8,9,10,11,12].

Population initialization methods also play a significant part in enhancing the performance of any population-based algorithm. Teo [13] proposed an exploring dynamic self-adaptive populations method for DE. Rahnamayan et al. [14] suggested an opposition-based method to initialize population for DE. Later, some noticeable works on population initialization methods and population size adaptation have been completed in [15,16,17,18,19,20,21].

A crossover and selection operation-based modifications given in [22,23,24] are also completed to enhance the performance of DE.

Other modifications include development of novel mutation techniques and their adaptive/self-adaptive strategies. Some modified mutation based DE methods are Trigonometric DE (TDE) [25], donor mutation DE (DNDE) [26,27], DE with random localization (DERL) [28], DE with hybrid mutation [29], DE with external archive (JADE) [30], DE with neighborhood mutation (DEGL) [31], Proximity-based mutation operators for DE [32], DE with modified random localization (MRLDE) [33], interpolation rules based mutation [34], ranking based mutation strategy [35], DE with multiple mutation strategies [36], iLSHADE [37], random perturbation based DE [38], IMODE [39], HiPDE [40], and so on.

Several research studies are carried out in the development of adaptive mutation strategies. Some recent research regarding adaptive DE is as follows. Qin and Suganthan [41] proposed SaDE to implement two mutation strategies *DE/rand/1*” and *DE/current-to-best/1* simultaneously. In SaDE the trial vector was created by either success ratios based strategy in the last 50 generations by using adaptive probability. Later, Qin et al. [42] extended this work for four mutation strategies. In their proposed work, both control parameter values and trial vector creation strategies are regularly self-adapted by learning from their prior experiences of the solutions. In SaDE, an adaptive rule is proposed for updating the probability of each variant according to their corresponding success or failure in performance.

Gong et al. [43] proposed a strategy adaptation approach based on the probability matching technique being fed by comparative fitness upgrading. They have also suggested diverse categories of strategy adaptation methods in which a strategy parameter is utilized to manage the selection of different strategies, and two straightforward strategy adaptation approaches are employed to revise the parameter. Later on, Mallipeddi et al. [44] proposed a new variant, EPSDE, with a collection of different mutation strategies and parameters. In this variant each mutation strategy is started randomly, and any mutation strategy is stored to the next generation for which the produced trial vector gives better fitness values than its target vector. Otherwise, it is selected randomly from the preceding winning strategies stored with identical probability. Some other strategy adaptation based DE variants are SspDE [45], composite DE (CoDE) [46], MDE_pBX [47], ISADE [48], Adaptive DE [49], and TS-MSCDE [50]. Various studies on the changes and applications of DE have been conducted periodically. Several of these excellent studies are documented in [51,52,53].

Apart from modifications in basic operations, a lot of research has also been completed to enhance the local search capability and introduce some additional features. Some of these recently developed variants are LSDE [54], DEGOS [55], CJADE [56], PAIDDE [57], TRADE [58] and so on.

In this study, a self-adaptive technique named as SaMDE has been developed in an effort to continue boosting the performance of DE. This method integrates MRLDE, DNDE, and SPIDE algorithms and takes advantage of all of their benefits in a single location. The selection of these tactics is motivated by their performance. These tactics have demonstrated excellent performance in the past, and we anticipate that their hybridization will further improve the performance of the proposed scheme. SaMDE begins with the provision of a systematic and superior way for selecting individuals for mutation operation by MRLDE, followed by the implementation of DNDE and SPIDE by the proposed self-adapting technique. The advantage of this technique is that if one variant fails, the other variant will automatically activate, and the algorithm will continue to run until the reaches the desired outcomes.

The rest of the paper is organized as follows: In the second section, a brief description of DE, MRLDE, DNDE, and SPIDE is offered. The SaMDE methodology is discussed in Section 3. Section 4 defines the benchmark test tasks and potential energy problem. In Section 5, the numerical and statistical results are analyzed and discussed. Section 6 concludes the entire investigation.

## 2. Background

### 2.1. Differential Evolution Algorithm

Similar to other EAs, DE also starts with a uniformly distributed set of solutions called population within a bound domain [*X_low_*, *X_upper_*]. Let at any generation *g*, the population set is $P^{\left(g\right)}=\{X_{i}^{\left(g\right)}\left|i=1,\;2,\;\cdots ,\;NP\}\right.$ where each $X_{i}^{\left(g\right)}=\{x_{i,\;j}^{\left(g\right)}:i=1,\;2\ldots NP;\;j=1,\;2,\ldots d\}$ is a *d*-dimensional vector and *NP* is the size of population, then it can be initially generated by Equation (1).

(1) $$X_{i}^{\left(g\right)}=X_{low}+rand(0,1)\times (X_{upper}-X_{low})$$

Next, the evolution phase starts where new positions for each individual are generated through mutation and crossover operation and then selection operation is applied to choose the best fitted vector to the next generation population. A DE algorithm can be written as *DE/a/b/c*, where *a*, *b* and *c* represent the number of vector differences, mutation and crossover strategy, respectively. There are several mutation techniques for DE, however we have utilized solely the *DE/rand/1/bin* strategy throughout this investigation. The mutation and crossover strategies for *DE/rand/1/bin* are defined as below.

*Mutation*: For any vector $X_{i}^{\left(g\right)}$, the mutant vector $M_{i}^{\left(g+1\right)}$ is generated through Equation (2).

(2) $$\;M_{i}^{\left(g+1\right)}=X_{r}^{\left(g\right)}+F\times (X_{s}^{\left(g\right)}-X_{t}^{\left(g\right)})$$

where $X_{r}^{\left(g\right)}$, $X_{s}^{\left(g\right)}$ and $X_{t}^{\left(g\right)}$ are three mutually different vectors randomly chosen from $P^{\left(g\right)}$ different from $X_{i}^{\left(g\right)}$. The vector $X_{i}^{\left(g\right)}$ is called as the base vector and F∈(0,1] is a parameter which is used to control the amplification of the variation $\left(X_{s}^{\left(g\right)}-X_{t}^{\left(g\right)}\right)$.

*Crossover:* Crossover operation is required to create a trail vector say $U_{i}^{\left(g+1\right)}=\{u_{i,\;j}^{\left(g+1\right)}:\;j=1,\;2,\ldots d\}$ by crossing the components of target vector $X_{i}^{\left(g\right)}=\{x_{i,\;j}^{\left(g\right)}:j=1,\;2,\ldots d\}$ and mutant vector $M_{i}^{\left(g+1\right)}=\{m_{i,\;j}^{\left(g+1\right)}:j=1,\;2,\ldots d\}$ by Equation (3).

(3) $$u_{i,j}^{\left(g+1\right)}=\left\{\begin{array}{ccc} m_{i,j}^{\left(g+1\right)} & if & rand_{j}(0,1)\leq C_{R}(OR)\;j\in randi(d)\\ x_{i,j}^{\left(g\right)} & & otherwise \end{array}\right.$$

where $C_{R}\in (0,\;1)$ known as the crossover parameter and *randi* (*d*) denotes the random index *j* from {1, 2,…*d*} which insures that at least one component in the trail vector should be chosen from the mutant vector.

*Selection*: This procedure selects the optimal vector from the target and trail vectors for the next generation population based on their fitness value as determined by Equation (4).

(4) $$X_{i}^{\left(g+1\right)}=\left\{\begin{array}{ccc} U_{i}^{\left(g+1\right)} & if & fun\;(U_{i}^{\left(g+1\right)})\leq fun(X_{i}^{\left(g\right)})\\ X_{i}^{\left(g\right)} & else & \end{array}\right.$$

### 2.2. Mutation Based Modified DE Variants

The primary objective of the mutation operation is to provide a new position for every randomly selected vector by adding it to the weighted difference of two different vectors. The vector that must be perturbed is known as the base vector, while the other two are known as the difference vectors. According to Kaelo and Ali [28], the newly created mutant vector is dependent on the nature of the base vector; hence, a suitable selection of the base vector may help to increase the convergence rate of the algorithm. Inspired by this concept, we have previously presented three new modified DE variants, DNDE [27], MRLDE [33], and SPIDE [34], in which the base vector is taken in an improved manner rather than randomly from the population.

This section will now provide a concise description of these variants.

#### 2.2.1. MRLDE

A modified version of the DERL algorithm was proposed in 2012 [33], and it is known as modified randomized localization-based DE (MRLDE). The choice of random vectors to carry out the mutation is the only distinction between MRLDE and DE. MRLDE divides the entire population into three segments, say, $P_{best}^{\left(g\right)}$, $P_{medium}^{\left(g\right)}$ and $P_{worst}^{\left(g\right)}$ of size λ_1_, λ_2_ and λ_3_ by the fitness values and then select the vectors $X_{r}^{\left(g\right)}$, $X_{s}^{\left(g\right)}$ and $X_{t}^{\left(g\right)}$ from the $P_{best}^{\left(g\right)}$, $P_{medium}^{\left(g\right)}$ and $P_{worst}^{\left(g\right)}$, respectively, to run the mutation operation as defined in the DE algorithm. The effectiveness of the algorithm has been implemented in some real-life problems such as image enhancement [59], economy load dispatch problem [60] and noise source identification [61].

#### 2.2.2. Sequential Parabolic Interpolation Based DE (SPIDE)

The concept of selecting a base vector in this form is inspired by sequential parabolic interpolation (SPI), a root finding approach for the equation *q*(*x*) = 0. If *x*_1_, *x*_2_ and *x*_3_ are three points with the function values *q*(*x*_1_), *q*(*x*_2_)) and *q*(*x*_3_), respectively, then the next root estimation by the SPI method is given by Equation (5).

(5) $$x_{4}=x_{1}+\frac{1}{2}\frac{{\left(x_{1}-x_{2}\right)}^{2}\{q(x_{1})-q(x_{3})\}-{\left(x_{1}-x_{3}\right)}^{2}\{q(x_{1})-q(x_{2})\}}{\left(x_{1}-x_{2})\{q(x_{1})-q(x_{3})\}-(x_{1}-x_{3})\{q(x_{1})-q(x_{2})\right\}}$$

In SPIDE, we replace *x*_1_, *x*_2_ and *x*_3_ by $X_{r}^{\left(g\right)}$, $X_{s}^{\left(g\right)}$ and $X_{s}^{\left(g\right)}$, respectively, in Equation (5) and generate a new vector say $X_{Q}^{\left(g+1\right)}$. Next we select the base vector among $X_{Q}^{\left(g+1\right)}$ or $X_{tb}^{\left(g\right)}$ by settinga probability (*P_s_*) where $X_{tb}^{\left(g\right)}$ denotes the best vector among $X_{r}^{\left(g\right)}$, $X_{s}^{\left(g\right)}$ and $X_{s}^{\left(g\right)}$. As a next estimation root, $X_{Q}^{\left(g+1\right)}$ gives a minimum fitness value than $X_{r}^{\left(g\right)}$, $X_{s}^{\left(g\right)}$ and $X_{s}^{\left(g\right)}$ and hence helps to increase the algorithm’s convergence speed. Refer to [34] to understand more about SPIDE and its operation.

#### 2.2.3. Donor Mutation Based DE (DNDE)

Fan et al. [26] suggested that the base vector can be taken as a weighted mean of selected vectors $X_{r}^{\left(g\right)}$, $X_{s}^{\left(g\right)}$ and $X_{s}^{\left(g\right)}$. We have used this idea and selected the base vector from these random vectors based on random localization approach [28] and weighted mean as suggested by [26] and named this variant as ‘DNDE’. In this approach, the weighted vector $X_{w}^{\left(g+1\right)}$ is generated by convex combination of three randomly selected vectors $X_{r}^{\left(g\right)}$, $X_{s}^{\left(g\right)}$ and $X_{s}^{\left(g\right)}$ by Equation (6).

(6) $$X_{w}^{\left(g+1\right)}=\nu_{1}X_{r}^{\left(g\right)}+\nu_{2}X_{s}^{\left(g\right)}+\nu_{3}X_{t}^{\left(g\right)}$$

Here $\nu_{i}$ = 1, 2, 3 are uniform random numbers in the range of (0, 1) and should satisfy the condition $\sum_{i}\;\nu_{i}=1$.

Now similar to SPIDE, the base vector is selected among $X_{w}^{\left(g+1\right)}$ or $X_{tb}^{\left(g\right)}$ by setting a probability (*P_d_*). In addition, [27] provides a comprehensive discussion of DNDE and its efficacy. The SPIDE and DNDE pseudo codes are listed in Algorithm 1.
**Algorithm** **1** *SPIDE* and *DNDE**For i* = 1 to *NP do*           Select three vectors $X_{r}^{\left(g\right)}$$X_{s}^{\left(g\right)}$ and $X_{t}^{\left(g\right)}$ such that $X_{r}^{\left(g\right)}\neq X_{s}^{\left(g\right)}\neq X_{t}^{\left(g\right)}\neq X_{i}^{\left(g\right)}$ And find best vector $X_{tb}^{\left(g\right)}$ between these vectors by their fitness           /∗∗∗∗∗∗∗*base vector by SPIDE* ∗∗∗∗∗∗∗/ Obtained $X_{Q}^{\left(g+1\right)}$ by Equation (5)           *if* (*rand* (0,1) < *P_s_*) $X_{r}^{\left(g\right)}=X_{Q}^{\left(g+1\right)}$
            *Else*
                   $X_{r}^{\left(g\right)}=X_{tb}^{\left(g\right)}$ *end if* /********************************/         /******base vector by DNDE ******/         Obtained $X_{w}^{\left(g+1\right)}$ by Equation (6)         *if* (*rand* (0,1) < *P_d_*) $X_{r}^{\left(g\right)}=X_{w}^{\left(g+1\right)}$
         
*Else*
                
$X_{r}^{\left(g\right)}=X_{tb}^{\left(g\right)}$
         *end if* /*****************************/         Perform mutation, Crossover and Selection *end for*

## 3. Proposed Self Adaptive Approach (SaMDE)

As noted by numerous researchers, the mutation strategies are significantly reliant on the problems under consideration. A substantial amount of time may be required to solve a single problem by attempting numerous ways. This dilemma motivated us to create a Self-adaptive mutation approach for DE (SaMDE) that can handle difficulties more effectively.

Proposed SaMDE is a fusion of MRLDE, DNDE and SPIDE. As recommended by the MRLDE method, the entire search space is initially partitioned into three sub-regions, and then the DNDE and SPIDE algorithms are utilized adaptively. The proposed rule updates the probability for mutation schemes based on their performance in any generation. Similar to a concept described by Qin et al. [41,42] in SaDE, where probability rules are updated based on the success and failure ratio of the variants, and then any variant is activated based on its probability. Instead of random activation, the variant with the highest likelihood or success rate will be activated in SaMDE.

In SaMDE, probabilities are initially assigned at random to each variant of DNDE and SPIDE, and then the evaluation procedure begins for the variant with the highest probability. This version shall be referred to as active variant.

Now, the active variant is assigned a positive rank if the trial vector created by this technique is picked for the following generation; otherwise, a negative rank is assigned. At the conclusion of a generation, all positive and negative ranks for the active variant are added together. Let *p*_1_ and *p*_2_ represent the probabilities for DNDE and SPIDE, respectively, and let *RP* and *RN* represent the total positive and negative rankings in any generation. The probability is then updated based on the following rules:

*Case 1*: When $p_{1}\geq p_{2}$, i.e., DNDE is active:

(7) $$p_{1}=\frac{RP}{RP+RN};\;\;\;\;\;p_{2}=\frac{RN}{RP+RN}$$

*Case 2*: When $p_{2}>p_{1}$, i.e., SPIDE is active:

(8) $$p_{2}=\frac{RP}{RP+RN};\;\;\;\;\;p_{1}=\frac{RN}{RP+RN}$$

These criteria assist in updating the probability of a self-adaptive procedure. If the active version performs better in a generation, it will be used in the following generation as well; otherwise, another variant will become the active variant. The updating criteria is immediately influenced by the rejection and acceptance performance (or acceptance rate) of trial vectors into the subsequent generation.

For example, if the acceptance rate for trial vectors generated by the active DE variant is better than its rejection rate then it will imply that the total number of positive ranks is higher than the total negative ranks. Therefore, the corresponding probability for the active variant will be high giving it a chance to continue in the next generation.

**Figure 1:**
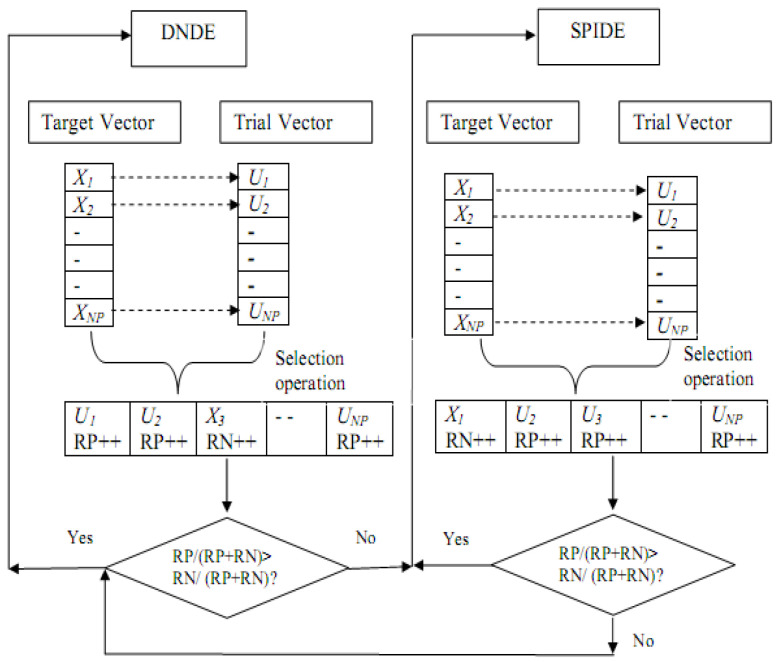
Working structure of proposed Self Adaptive Approach.

Similarly, a higher rejection rate of trial vectors increases the negative ranks decreasing the probability of the current active variant to be continued in the next generation. The operation of SaMDE is also illustrated in Figure 1.

It can also be noticed that for any generation, the total number of positive and negative rank should be equal to the population size, i.e., *RP* + *RN* = *NP*

Hence the rules (7) and (8) may be further reduced as follows:

(9) $$Case\;1:\;p_{1}=\frac{RP}{NP},\;p_{2}=1-p_{1}$$

(10) $$Case\;2:\;p_{2}=\frac{RP}{NP},\;p_{1}=1-p_{2}$$

Under the aforementioned rules, only the positive ranks of active variations should be considered. This will also reduce the amount of time spent counting negative ranks.

**Figure 2:**
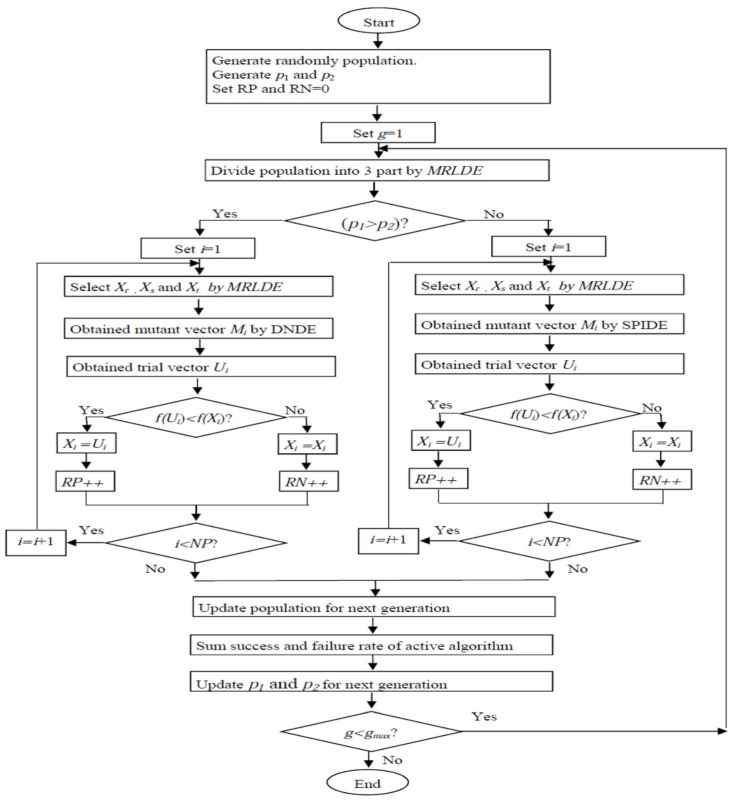
Flow chart of SaMDE.

By using SaMDE, the advantage of all three variants MRLDE, DNDE and SPIDE are considered in a single algorithm. First of all, MRLDE provides a strategic method for selecting the individuals for mutation which creates a platform for getting a fast convergence speed. Secondly, DNDE and SPIDE are employed adaptively to make SaMDE more efficient. By using the adaptive rule, the variant which gives successively better performance obtains additional chances to be continued for the next generations. Hence if any variant fails to solve a specific problem the other variant will be automatically activated to solve it. However, a drawback of SaMDE is, if neither of the variants are able to solve a specific problem, performance of SaMDE will naturally deteriorate. Next, the flowchart of the suggested adaptive strategy and pseudo code for SaMDE is depicted in Figure 2 and Algorithm 2, respectively.
**Algorithm 2** *SaMDE*Set *NP, F* and *C_R_*, *p*_1_ and *p*_2_Generate population *P^G^* = {*X_i_^(g)^*, *i* = 1, 2,..., *NP*}.         Evaluate *f(X_i_^(g)^)* and Sort whole population by their fitness i.e., *Sort* {*f(X_i_^(g)^)*}         Generate probability *p*_1_ and *p*_2_ randomly and set positive and negative rank *RP* = *RN* = 0         ***while*** (Termination criteria is not satisfied) ***do***               ***for** i* = 1 to *NP **do***                      Select *r, s* and *t* by *MRLDE* for each *i*                      ***If** p*_1_ > *p*_2_                                execute mutation and crossover by DNDE                                ***if*** trial vector selected for the next generation                                         Increase a positive rank i.e., *RP* = *RP* + 1                                ***else***
                                         Increase a negative rank i.e., *RN* = *RN* + 1                                ***end if***                      ***else***                                execute mutation and crossover by by SPIDE                                ***if*** trial vector selected for the next generation                                         Increase a positive rank *RP* = *RP* + 1                                ***else***                                         Increase a negative rank *RN* = *RN* + 1                                ***end if***                      
***end if***
               ***end for***               Update the population for the next generation, *P^g+1^ =* {*X_i_^(g+1)^*, *i* = 1,2,...*NP*}               *Sort* {*f(X_i_^(g+1)^)*}               Update *p*_1_ and *p*_2_ using Equations (7) and (8)         
***end while***


## 4. Test Problems and Real-Life Application

### 4.1. Test Suit-1: Classical Benchmarks Problems

In the first test suite there are 15 traditional benchmark problems selected from various literature [4,14,30,42,43]. All these problems are tested for dimension 30. The test functions *f*_1_–*f*_5_, *f*_14_ and f_15_ are unimodal, *f*_6_ is a discontinuous function with one minimum, *f*_7_ is a noisy function. The test functions *f*_8_–*f*_13_ are multimodal functions in which the number of local minima increases exponentially with the problem dimension [4]. According to literature unimodal functions are important to check the exploration ability and convergence rate of algorithms while multimodal functions are important to check the exploitation ability of algorithm.

### 4.2. Test Suit-2: IEEE CEC2008 Functions

The second set consists of six shifted functions (*SF*_1_–*SF*_6_) selected from the IEEE CEC 2008 test suit [62]. This test suite was particularly planned to test the efficiency and robustness of an algorithm on complex test problems.

A review of classical and CEC2008 benchmark functions is presented in Table 1.

### 4.3. Test Suit-3: IEEE CEC 2017 Functions

The IEEE CEC 2017 test suit is renowned as a set of extremely complex benchmark functions. There is a total of 29 benchmark (C_1_–C_30_) problems in the suit where one function C_2_ has been removed due to its high unstable nature. These functions can be classified in four categories like Unimodel functions (C_1_–C_3_), Multimodel functions (C_4_–C_10_), hybrid functions (C_11_–C_20_) and composite functions (C_21_–C_30_). The bound for the variable for all functions is (−100, 100) and the optimum value is 100×i where ‘*i*’ is function number from [1,30]. A detailed review and specification of CEC 2017 functions can be found in [63].

### 4.4. Real Life Application: Molecular Potential Energy Problem

The minimization of the potential energy problem of a molecule is a complex and multimodal optimization problem that occurs in the chemical science field [27,64,65]. The most challenging aspect of this problem is that the number of local minimizers grows exponentially as the size of the molecule rises. Consequently, it becomes a difficult and unsolved challenge for scientists and engineers from a variety of disciplines.

The mathematical model of a molecular having a linear chain of n-beads centred at x_1_, x_2_,_._...x_n_ in a 3D domain. The optimization model of molecular potential energy is given as below:

(11) $$\mathrm{Minimize}\;F=\sum_{i=1}^{4}F_{i}$$

where *F*_1_, *F*_2_, *F*_3_ and *F*_4_ are the potential forces due to bond length, bond angle, torsion angles and interaction, respectively, and defined as below:

(12) $$\begin{array}{l} F_{1}=\sum_{(i,j)\in K_{1}}s_{i,j}^{1}{\left(\rho_{i,j}-\rho_{i,j}^{0}\right)}^{2}\\ F_{2}=\sum_{(i,j)\in K_{2}}s_{i,j}^{2}{\left(\phi_{i,j}-\phi_{i,j}^{0}\right)}^{2}\\ F_{3}=\sum_{(i,j)\in K_{3}}s_{i,j}^{3}(1+\cos(3\theta_{i,j}-\theta_{i,j}^{0}))\\ F_{4}=\sum_{(i,j)\in k_{3}}\left(\frac{{\left(-1\right)}^{i}}{\rho_{i,j}}\right) \end{array}$$

Here $\rho_{i,j}$$\phi_{i,j}$ and $\theta_{i,j}$ are bond length, bond angle and torsion angle between two, three and four consecutive pairs ofbeads, respectively. *s*^1^*_i,j_*, *s*^2^*_i,_*_j_, and *s*^3^*_i,j_* denotes bond stretching, angle bending and the torsion force constant, respectively. *K_j_*, *j* = 1, 2, 3 denotes the set of pair of atoms separated by *j*-covalent bonds.

As explained in [64], the final optimization function can be defined by Equation (13):

(13) $$Min\;F=\sum_{i=1}^{n-32}\left((1+\cos(3\theta_{i,\;i+3}))+\frac{{\left(-1\right)}^{i}}{\sqrt{10.60099896-4.141720682\;\cos}(\theta_{i,i+3})}\right)$$

The optimization model in Equation (13) is a non-convex function and has several local minimizers even for the small value of *n*. The number of local minimizers of the function is 2^n−3^, and the doamin bound for *θ_i,j_;* will be restricted in (0, 5).

## 5. Result Analysis and Discussion

### 5.1. Experimental Settings:

Experiments are conducted using a 64-bit equipped laptop of Dell company with a 2.6-GHz Intel Core i3 processor, 8 GB RAM, and Windows 10 operating system. Other parameters settings were taken as suggested in various literature [27,33,34,42,43]:

- NP = 100; D = 30,50; F = 0.5; Cr =0.9.
- For MRLDE, λ_1_ = 20, λ_2_ = 40 and λ_3_ = 40 [33].
- For SPIDE and DNDE, P_s_ = 0.1, P_d_ = 0.1 [27,34].
- Max NFE = 10,000 D for all functions.
- Total Run = 50.

### 5.2. Performance Evaluation of SaMDE over DE, SPIDE, DNDE, MRLDE

#### 5.2.1. Results Analysis in Terms of Average Error and Standard Deviation:

The experimental results for traditional functions (*f*_1__–_*f*_15_) and the shifted function (*SF*_1_–*SF*_6_) are presented in Table 2. When many algorithms can obtain the global optima, then only intermediate solutions for the function are presented.

From Table 2, it is clear that all modified variants give superior performance over basic DE for all functions, except DNDE in case of function *f*_4_. SaMDE has obtained best results in case of all functions except 03 functions *f*_2_, *f*_5_ and *f*_8_ where MRLDE is in the leading position. MRLDE obtained the second best position on 09 functions while SPIDE obtained the second position in the case of 03 functions *f*_4_, *f*_7_, *f*_9_. Similarly, DNDE also obtained the second position in the case of 03 functions *f*_12_, *f*_13_ and *SF*_4_. In case *SF*_1_, both SaMDE and DNDE gives equal accuracy and obtains the first position. Similarly, SaMDE, MRLDE and gives DNDE equal performance on *SF*_6_. In the case of *SF*_5_, all four variants give equal performances.

A statistical analysis based on the mean difference of samples is also conducted in Table 2. The symbols ‘+’, ‘−’ and ‘=’ show the performance of SaMDE as significantly better, worse or equal, respectively, with its competitor. As per the last column of table, the win/loss/tie performance of SaMDE is 21/0/0, 20/0/1, 18/0/3 and 17/2/2/ with respect to DE, SPIDE, DNDE and MRLDE, respectively, ×10^−^/×10^+^.

#### 5.2.2. Result Analysis by Non-Parametric Statistical Tests

Three non-parametric statistical tests ‘Wilcoxon rank sum test’, ‘Friedman rank test’ and ‘Bonferroni-Dunn test’ [66] are used to check the significant difference between the performances of the proposed variants. These results are tabulated in Table 3 and Table 4.

The Wilcoxon rank sum test results are given in Table 3. ΣR^+^ and ΣR^−^ represent the sum of ranks for positive and negative differences, respectively. A higher positive rank sum shows the SaMDE over other algorithms. The z-value and corresponding *p*-value are also given in Table 3. The significant level of difference is taken as α = 0.05. From this table, it can be noticed that all variants obtained significantly better results in comparison to DE. MRLDE gives better results than SPIDE whereas the performances of MRLDE and DNDE are significantly equal. SaMDE gives the best performance in comparison to other variants.

Attending the results given in Table 4, the Friedman’s rank test and Bonferroni–Dunn’s test are used to detect significant differences for the control algorithm SaMDE and the results are presented in Table 4.

**Figure 3:**
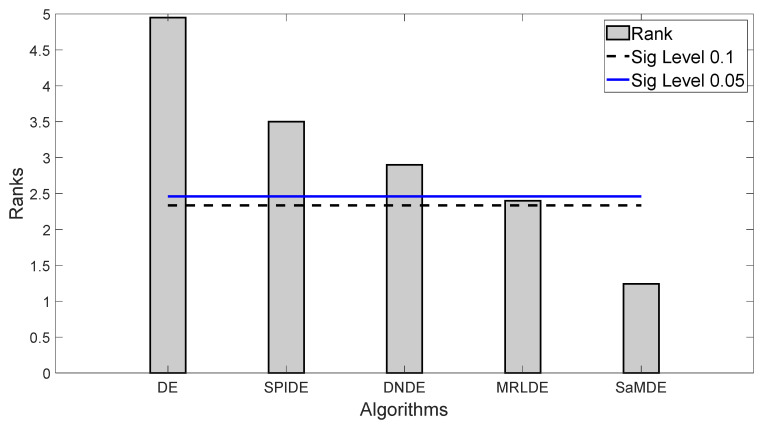
Bonferroni–Dunn bar chart for DE, SPIDE, DNDE, MRLDE and SaMDE. The Bar represents the algorithm’s rank and Horizontal cut lines represents the significant levels.

It can be noticed that SaMDE obtained the lowest mean rank among all other variants. In Figure 3, Bonferroni–Dunn’s graphic demonstrates the difference between the rankings of each algorithm. The algorithm with the lowest rank is considered as the control algorithm, while the horizontal cut line represents the threshold for the control algorithm. This line is drawn at a distance of the sum of the ranking of the control algorithm and the corresponding CD calculated by the Bonferroni–Dunn method as shown in Table 4 for each α = 0.1 and α = 0.05. The algorithms for which the rank bar exceeds this line are considered to have a worse performance than the control algorithm. Hence, by using the application of the Bonferroni–Dunn method, it can also be seen that only MRLDE is significantly acceptable when compared to SaMDE.

#### 5.2.3. Performance Evaluation of SaMDE by Convergence Curves

**Figure 4:**
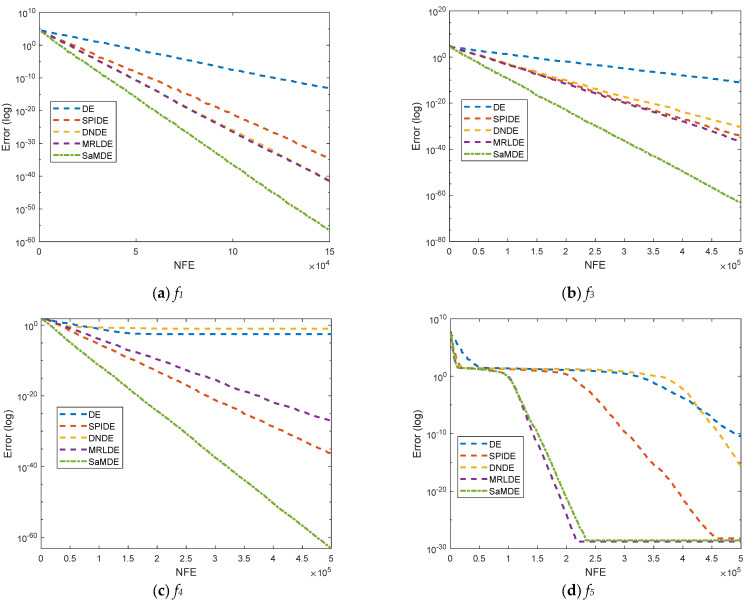

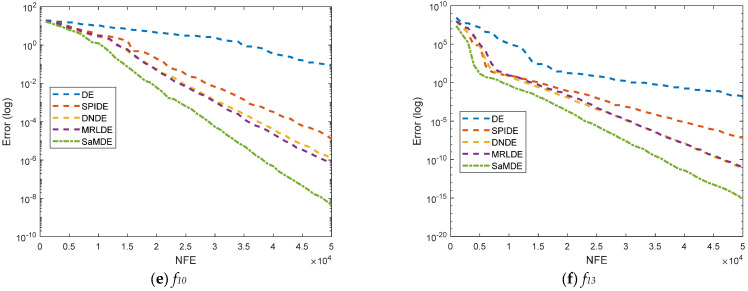

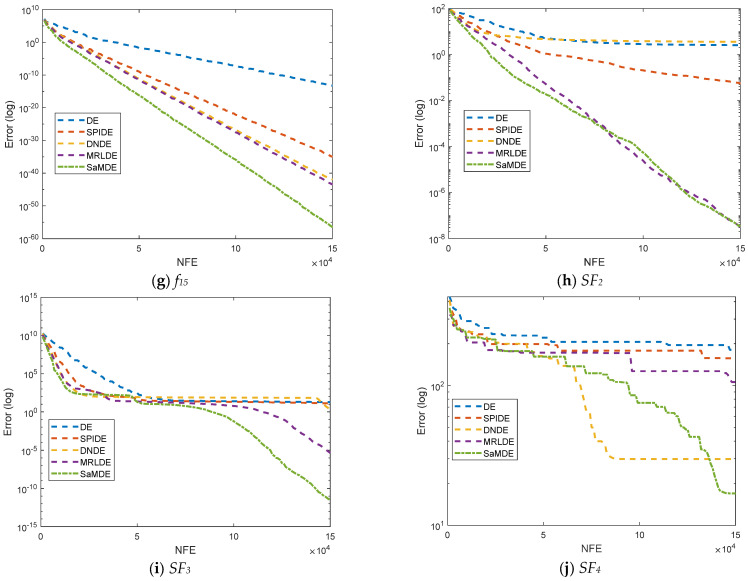
Convergence graphs for functions *f*_1_, *f*_3_, *f*_4,_ *f*_5_, *f*_10_, *f*_13_, *f*_15_, *SF*_2_, *SF*_3_ and *SF*_4_.

Convergence curves for DE, SPIDE, DNDE, MRLDE and SaMDE of selected functions such as *f*_1_, *f*_3_, *f*_4_, *f*_5_, *f*_10_, *f*_13_, *f*_15_, *SF*_2_, *SF*_3_ and *SF_4_* are represented in Figure 4. The X-axis and Y-Axis represents the NFE and its corresponding error value, respectively. By the graphs it can be easily seen that SaMDE performs faster and confirms its robustness over its parent variants.

### 5.3. Performance Evaluation of SaMDE over Other Enhanced DE Variants

In this section the performance of SaMDE is compared with SHADE [5], JADE [30], SaDE [42], rJADE [43], APadapSS-JADE [43], and DEGOS [55] algorithms. The comparison is taken in terms of average error and standard deviation and then non-parametric tests are applied to check the significant difference between the algorithms. The results for SHADE are taken from [5], for SaDE and JADE are taken from [30], and for rJADE and APadapSS-JADE are taken from [43]. For DEGOS, original results have been obtained by using the code provided on [67]. In order to make a fair comparison, all parameter settings remain similar for all algorithms.

According to Table 5, SaDE, JADE, rJADE, APadapSS-JADE, SHADE, DEGOS and SaMDE have obtained the best results on 0, 0, 1, 5, 2, 0 and 5 functions, respectively. From the win/loss/tie row of the table, we can see that SaMDE surpasses the algorithm SaDE, JADE, rJADE, APadapSS-JADE, SHADE and DEGOS on 12, 10, 11, 6, 7 and 12 cases, respectively.

The Wilcoxon rank sum test presented in Table 6 shows that SaMDE performs significantly equal with respect to APAdapSS-JADE and SHADE while it is significantly better than SaDE, JADE, rJADE and DEGOS.

Table 7 shows the results of the Friedman test and the Bonferroni–Dunn test for the results given in Table 7 in order to check the global significant difference between the algorithms. SaMDE obtained the lowest rank and hence proves its significance over others.

**Figure 5:**
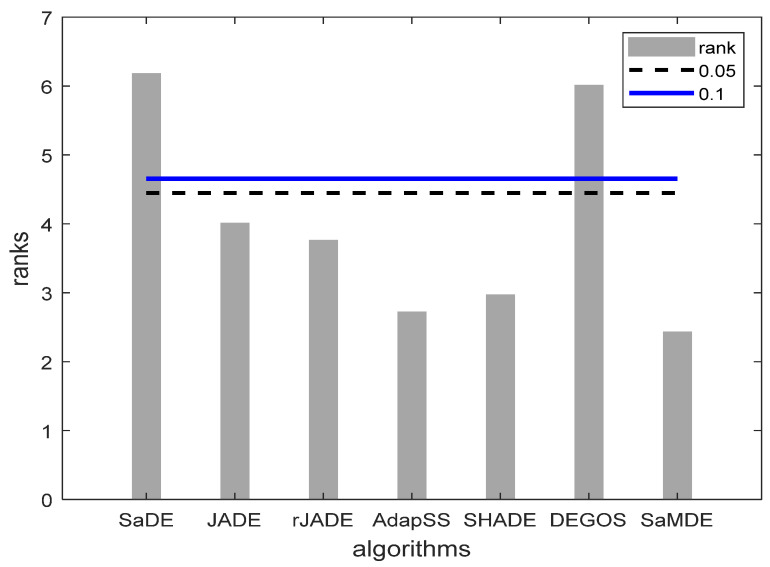
Bonferroni–Dunn bar charts on the results as given in Table 7. The Bar represents the algorithm’s rank and horizontal cut lines represent the significant levels.

Figure 5 represents the bar graphs of rank of the algorithms. The horizontal control lines are drawn for a significant level at α = 0.1 and α = 0.05 by taking by taking SaMDE as a control algorithm. The graph shows that the ranks of SaMDE, JADE, rJADE, AdapSS-JADE and SHADE lie under the control lines of the significant level at α = 0.1 and α = 0.05. Hence, these algorithms can be considered as significantly equal with SaMDE and significantly better than SaDE and DEGOS.

### 5.4. Performance Evaluation of SaMDE on CEC2017 Functions

In this section a performance evaluation of SaMDE has been tested on more complex and widely used benchmark functions taken from the IEEE CEC2017 test suit. The performance of SaMDE has been compared with seven recent DE variants as SHADE [5], Deexp [24], iLSHADE [37], DEGOS [55], CJADE [56], PAIDDE [57] and TRADE [58]. We have also compared the performance with another metaheuristic algorithm, HMRFO [68], which is an enhanced variant of the recently developed Manta Ray Foraging Optimization Algorithm. The results for Deexp, iLSHADE are taken from [24], and for PAIDDE, DEGoS, CJADE, IMODE and SHADE are taken from [57]. For TRADE and HMRFO, the original results have been obtained by using the code given in [67]. The population size and maximum NFEs have been taken as 100 and 300,000, respectively, for all algorithms.

In Table 8, all results are collected in terms of average error and standard deviation of 50 runs. According to the table, SaMDE, TRADE, DEexp, iLSHADE, PAIDDE, DEGoS, CJADE, IMODE, SHADE and HMRFO have obtained the best results on 14, 7, 10, 4, 8, 3, 2, 1, 5 and 0 functions, respectively. From the win/loss/tie row of the table, we can see that SaMDE surpasses the algorithm TRADE, DEexp, ILSHADE, PAIDDE, DEGoS, CJADE, IMODE, SHADE and HMRFO on 19, 15, 17, 14, 24, 25, 27, 21 and 27 cases, respectively.

The Wilcoxon rank sum test is presented in Table 9. The higher sum of positive ranks shows the superiority of SaMDE over its competitors. However, the *p*-values show that the performance of DEexp, iLSHADE and PAIDDE will be considered as significantly equal with SaMDE, whereas SaMDE has a significant advantage over the rest of its competitors.

The results of the Friedman test and the Bonferroni–Dunn test on the CEC 2017 performance are given in Table 10. SaMDE obtained lowest rank and hence proves its significance over others.

**Figure 6:**
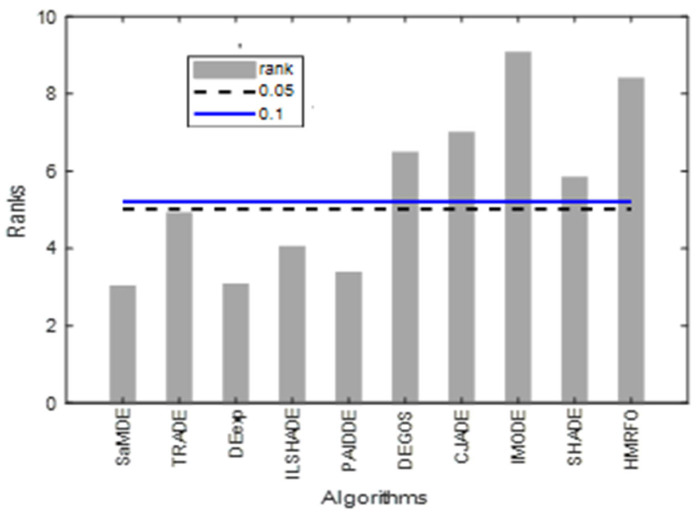
Bonferroni–Dunn bar charts on the results as given in Table 10. The Bar represents the algorithm’s rank and horizontal cut lines represent the significant levels.

Figure 6 represents the bar graphs of rank of the algorithms by taking SaMDE as a control algorithm. Graph shows that the ranks of SaMDE, TRADE, DEexp, iLSHADE and PAIDDE lie under the control lines of a significant level at α = 0.1 and α = 0.05 and hence these algorithms will be considered as significantly equal with each other and also significantly better than rest of the algorithms.

**Figure 7:**
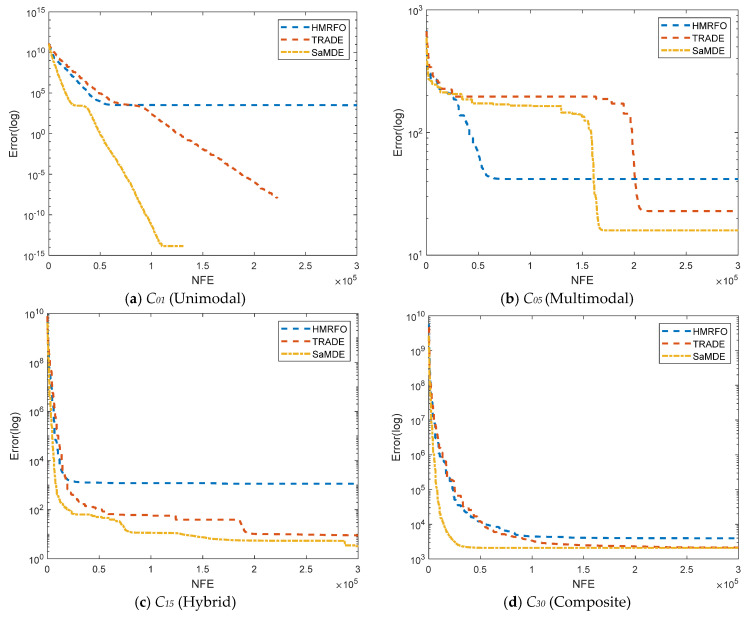
Convergence graphs for CEC-2017 functions: (**a**) *C*_01_ (**b**) *C*_05_ (**c**) *C_15_ and* (**d**) *C*_30_.

To analyze the convergence behavior of SaMDE, the convergence graphs of SaMDE with TRADE and HMRFO on selected functions C_1_, C_5_, C_15_ and C_30_ are represented in Figure 7. Graphs confirm the robustness of SaMDE to achieve the accuracy rapidly.

### 5.5. Performance Evaluation of SaMDE on Molicular Potential Energy Problem

The experimental results for DE, TDE, DERL, MRLDE and SaMDE are given in Table 11. In order to make a fair comparison, similar, all parameter settings are used for each algorithm as given in previous section. The experiments are executed for *n* = 10, 15, 20 and 25 where *n* indicates the number of beads. The results are obtained in terms of best, worst and mean fitness value and standard deviation (SD) on 50 runs.

It can be seen that for *n* = 10, the fitness value obtained by SaMDE is −0.589389 which is best among those obtained by other algorithms. It can also be noticed that all algorithms perform similarly in terms of best fitness value. MRLDE gives the second-best performance for *n* = 10. Similarly, it can be seen that SaMDE gives the best results for *n* = 15, 20 and 25. MRLDE gives the second best performance while DERL and TDE take third and fourth place, respectively, for each *n* = 15, 20 and 25.

**Figure 8:**
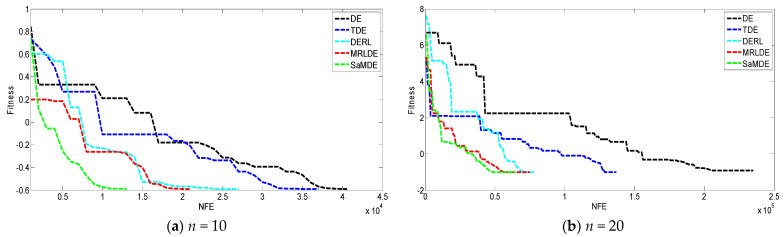
Convergence graphs of molecular potential energy problem for (**a**) *n =* 10, (**b**) *n =* 20.

Figure 8 represents the convergence graphs’ potential molecular energy problem at *n* = 10 and 20. The X-axis and Y-axis represent the NFE and its corresponding error value, respectively. By the graphs it can be easily seen that SaMDE performs faster and confirms its robustness over other variants.

## 6. Conclusions

In this study, a novel self-adaptive method called SaMDE has been developed and used to MRLDE, a mutation-based enhanced DE variant. This method applies the MRLDE algorithm after selecting an appropriate mutation strategy from DNDE and SPIDE. The performance of the proposed approach has been investigated on 50 benchmark problems and on a real-life application in the potential molecular energy problem. The summary of the paper is given below:

- During the initial series of trials, the proposed SaMDE is compared against DE and its parent versions MRLDE, DNDE, and SPIDE. SaMDE provides the best performance among all versions, whereas MRLDE provides the second-best performance.
- In the second series of studies, the performance of SaMDE is compared to five other enhanced DE variations, namely SaDE, JADE, rJADE, APadapSS-JADE, SHADE and DEGOS. The comparison is made in terms of average error and standard deviation. The numerical results demonstrate that SaMDE outperforms every other algorithm. However, the non-parametric tests show that APadapSS-JADE and SHADE performs significantly equal to the SaMDE.
- The third series of experiments includes the performance evaluation of SaMDE on more complicated benchmark functions taken from the CEC 2017 test suit. The results of SaMDE have been compared with 07 other recent DE variants named SHADE, DEexp, iLSHADE, DEGOS, CJADE, PAIDDE, TRADE and one metaheuristic named HMRFO. The numerical and statistical results demonstrated that SaMDE was superior to all of the algorithms except DEexp, iLSHADE and PAIDDE whose performances are significantly equal to the SaMDE.
- Finally, the performance of SaMDE is validated to minimize the molecular potential energy. In this problem, the number of local minima increases exponentially with the dimension of the problem. Hence experiments are conducted on various size (*n* = 10, 15, 20 and 25) of molecular beads. Compared to DE, TDE, DERL, and MRLDE, SaMDE provides more exact results with a faster convergence rate, as shown by the results.

Future work will first concentrate on extending the SaMDE to tackle constrained and multi objective optimization problems. Second, utilizing the self-adaptive technique that has been suggested, it will be fascinating to hybridize different metaheuristics algorithms.

## Figures and Tables

**Table 1:** Benchmark functions with initial bounds and optimum value.

Table	Initial Bound	f(x*)	Test Function	Initial Bound	f(x*)
*f*_1_: Sphere	(−100; 100)	0	*f*_12_: Generalized Penalized-1	(−50, 50)	0
*f*_2_: Schwefel’s-2.22	(−10, 10)	0	*f*_13_: Generalized Penalized-2	(−50, 50)	0
*f*_3_: Schwefel’s 1.2	(−100; 100)	0	*f*_14_: Exponetial	(−1, 1)	0
*f*_4_: Schwefel’s-2.21	(−100; 100)	0	*f*_15_: Zhakarov	(−5, 10)	0
*f*_5_: Rosenbrock’s	(−30, 30)	0	*SF*_1_: Shifted Sphere	(−100; 100)	−450
*f*_6_: Step	(−100; 100)	0	*SF*_2_: Schwefel’s 2.21	(−100; 100)	−450
*f*_7_: Noise	(−1.28, 1.28)	0	*SF*_3_: Shifted Rosenbrock	(−100; 100)	390
*f*_8_: Schwefel’s2.26	(−500, 500)	0	*SF*_4_: Shifted Rastrigin	(−5.12, 5.12)	−330
*f*_9_: Rastrigin’s	(−100; 100)	0	*SF*_5_: Shifted Griewank	(−600, 600)	−180
*f*_10_: Ackley’s	(−32, 32)	0	*SF*_6_: Shifted Ackley	(−32, 32)	−140
*f*_11_: Griewank	(−600, 600)	0			

**Table 2:** Comparison of SaMDE with DE, SPIDE, DNDE and MRLDE in terms of best, mean values and standard deviation (SD) at D = 30.

Fun	Max-NFE	Error	Numerical Results					Statistical Significance			
			DE	SPIDE	DNDE	MRLDE	SaMDE	5/1	5/2	5/3	5/4
*f*_1_	150 k	Best	1.17 × 10^−14^	4.37 × 10^−36^	1.56 × 10^−43^	1.13 × 10^−43^	2.11 × 10^−57^	+	+	+	+
		Mean	4.03 × 10^−14^	4.64 × 10^−35^	4.42 × 10^−42^	1.17 × 10^−42^	4.75 × 10^−57^				
		SD	2.71 × 10^−14^	5.50 × 10^−35^	3.58 × 10^−42^	1.33 × 10^−42^	2.23 × 10^−57^				
*f*_2_	200 k	Best	9.21 × 10^−11^	1.21 × 10^−24^	6.83 × 10^−32^	1.41 × 10^−41^	3.56 × 10^−38^	+	+	+	−
		Mean	5.18 × 10^−10^	6.75 × 10^−24^	7.05 × 10^−31^	2.68 × 10^−41^	6.39 × 10^−38^				
		SD	3.46 × 10^−10^	4.52 × 10^−24^	2.81 × 10^−31^	1.19 × 10^−41^	3.52 × 10^−38^				
*f*_3_	500 k	Best	9.82 × 10^−12^	1.56 × 10^−36^	1.09 × 10^−31^	8.75 × 10^−38^	1.71 × 10^−63^	+	+	+	+
		Mean	1.11 × 10^−11^	2.30 × 10^−35^	6.89 × 10^−30^	2.72 × 10^−37^	5.43 × 10^−63^				
		SD	3.36 × 10^−12^	3.28 × 10^−36^	6.61 × 10^−30^	1.91 × 10^−37^	4.07 × 10^−61^				
*f*_4_	500 k	Best	2.49 × 10^−09^	1.22 × 10^−37^	9.87 × 10^−02^	1.21 × 10^−28^	2.67 × 10^−64^	+	+	+	+
		Mean	3.18 × 10^−01^	2.61 × 10^−37^	6.97 × 10^−01^	3.21 × 10^−27^	4.30 × 10^−64^				
		SD	6.25 × 10^−01^	2.99 × 10^−37^	6.09 × 10^−01^	2.34 × 10^−27^	8.70 × 10^−64^				
*f*_5_	500 k	Best	1.16 × 10^−13^	4.99 × 10^−28^	1.26 × 10^−20^	0.00 × 10^+00^	1.83 × 10^−30^	+	+	+	−
		Mean	3.32 × 10^−12^	1.72 × 10^−28^	5.07 × 10^−17^	1.65 × 10^−30^	2.13 × 10^−30^				
		SD	2.89 × 10^−12^	1.22 × 10^−29^	1.01 × 10^−16^	8.15 × 10^−30^	1.34 × 10_−30_				
*f*_6_	10 k	Best	1.69 × 10^+03^	4.80 × 10^+01^	7.50 × 10^+00^	1.00 × 10^+01^	1.00 × 10^+00^	+	+	+	+
		Mean	2.17 × 10^+03^	6.41 × 10^+01^	9.50 × 10^+00^	1.08 × 10^+01^	1.10 × 10^+00^				
		SD	2.49 × 10^+02^	1.32 × 10^+01^	1.21 × 10^−01^	4.00 × 10^−01^	4.89 × 10^−01^				
*f*_7_	300 k	Best	2.89 × 10^−03^	1.29 × 10^−03^	1.12 × 10^−03^	1.30 × 10^−03^	1.81 × 10^−04^	+	+	+	+
		Mean	5.81 × 10^−03^	1.47 × 10^−03^	5.13 × 10^−03^	1.56 × 10^−03^	3.08 × 10^−04^				
		SD	6.69 × 10^−03^	3.51 × 10^−04^	2.80 × 10^−04^	1.89 × 10^−04^	1.70 × 10^−04^				
*f*_8_	500 k	Best	5.61 × 10^+03^	5.72 × 10^+02^	3.35 × 10^+02^	2.17 × 10^+02^	2.36 × 10^+02^	+	+	+	+
		Mean	6.23 × 10^+03^	2.85 × 10^+03^	6.98 × 10^+02^	1.82 × 10^+03^	6.36 × 10^+02^				
		SD	4.44 × 10^+02^	1.74 × 10^+02^	3.05 × 10^+02^	1.44 × 10^+03^	3.31 × 10^+02^				
*f*_9_	500 k	Best	8.59 × 10^+01^	8.67 × 10^−18^	1.04 × 10^−14^	1.39 × 10^+01^	0.00 × 10^+00^	+	+	+	+
		Mean	9.32 × 10^+01^	9.54 × 10^−18^	3.97 × 10^−14^	1.61 × 10^+01^	0.00 × 10^+00^				
		SD	5.39 × 10^+00^	9.50 × 10^−19^	7.95 × 10^−14^	2.31 × 10^+00^	0.00 × 10^+00^				
*f*_10_	50 k	Best	1.83 × 10^−02^	1.02 × 10^−05^	5.16 × 10^−06^	7.21 × 10^−07^	1.02 × 10^−10^	+	+	+	+
		Mean	9.16 × 10^−02^	1.45 × 10^−05^	6.19 × 10^−06^	8.02 × 10^−07^	2.71 × 10^−10^				
		SD	2.81 × 10^−02^	3.54 × 10^−05^	1.02 × 10^−06^	1.12 × 10^−07^	5.53 × 10^−09^				
*f*_11_	50 k	Best	2.21 × 10^−02^	1.18 × 10^−09^	9.75 × 10^−11^	1.76 × 10^−12^	7.94 × 10^−17^	+	+	+	+
		Mean	5.35 × 10^−02^	8.78 × 10^−09^	1.75 × 10^−11^	3.38 × 10^−12^	2.94 × 10^−16^				
		SD	1.94 × 10^−02^	4.74 × 10^−09^	4.62 × 10^−11^	2.91 × 10^−11^	1.81 × 10^−16^				
*f*_12_	50 k	Best	2.11 × 10^−03^	1.45 × 10^−09^	4.74 × 10^−13^	3.84 × 10^−13^	1.18 × 10^−17^	+	+	+	+
		Mean	3.73 × 10^−03^	3.85 × 10^−09^	4.64 × 10^−13^	8.23 × 10^−13^	2.01 × 10^−17^				
		SD	1.36 × 10^−03^	2.51 × 10^−09^	1.40 × 10^−13^	6.72 × 10^−13^	2.16 × 10^−17^				
*f*_13_	50 k	Best	1.75 × 10^−02^	6.93 × 10^−08^	3.53 × 10^−13^	7.33 × 10^−12^	8.79 × 10^−17^	+	+	+	+
		Mean	3.68 × 10^−02^	1.93 × 10^−07^	4.08 × 10^−12^	1.41 × 10^−11^	1.29 × 10^−16^				
		SD	1.91 × 10^−02^	7.73 × 10^−07^	1.22 × 10^−12^	1.11 × 10^−11^	2.67 × 10^−16^				
*f*_14_	50 k	Best	2.27 × 10^−06^	2.38 × 10^−14^	2.49 × 10^−16^	1.08 × 10^−16^	2.16 × 10^−19^	+	+	+	+
		Mean	6.68 × 10^−06^	4.67 × 10^−14^	3.55 × 10^−16^	2.24 × 10^−16^	2.16 × 10^−19^				
		SD	5.98 × 10^−06^	1.33 × 10^−14^	1.21 × 10^−16^	1.24 × 10^−16^	0.00 × 10^+00^				
*f*_15_	150 k	Best	2.74 × 10^−14^	1.12 × 10^−35^	8.45 × 10^−43^	3.58 × 10^−44^	1.85 × 10^−57^	+	+	+	+
		Mean	3.33 × 10^−14^	2.02 × 10^−35^	1.65 × 10^−42^	4.51 × 10^−43^	2.68 × 10^−57^				
		SD	1.41 × 10^−14^	1.07 × 10^−35^	1.27 × 10^−42^	3.68 × 10^−43^	6.52 × 10^−57^				
*SF*_1_	150 k	Best	5.68 × 10^−14^	0.00 × 10^+00^	0.00 × 10^+00^	0.00 × 10^+00^	0.00 × 10^+00^	+	+	=	+
		Avg	4.43 × 10^−13^	2.27 × 10^−14^	0.00 × 10^+00^	1.13 × 10^−14^	0.00 × 10^+00^				
		SD	3.84 × 10^−13^	2.78 × 10^−14^	0.00 × 10^+00^	2.27 × 10^−14^	0.00 × 10^+00^				
*SF*_2_	150 k	Best	2.16 × 10^−01^	5.60 × 10^−02^	2.65 × 10^+00^	3.89 × 10^−08^	2.81 × 10^−08^	+	+	+	+
		Avg	9.22 × 10^+00^	2.39 × 10^−01^	4.55 × 10^+00^	4.45 × 10^−07^	9.76 × 10^−08^				
		SD	1.09 × 10^+00^	2.81 × 10^−01^	1.64 × 10^+00^	8.27 × 10^−07^	8.78 × 10^−08^				
*SF*_3_	150 k	Best	1.89 × 10^+01^	7.48 × 10^+00^	2.91 × 10^−01^	1.13 × 10^−08^	3.24 × 10^−12^	+	+	+	+
		Avg	1.95 × 10^+01^	1.13 × 10^+01^	9.80 × 10^+00^	4.34 × 10^−06^	5.23 × 10^−10^				
		SD	1.08 × 10^+00^	2.66 × 10^+00^	7.51 × 10^+00^	6.64 × 10^−06^	4.22 × 10^−10^				
*SF*_4_	150 k	Best	1.64 × 10^+02^	1.41 × 10^+02^	1.42 × 10^+01^	9.01 × 10^+01^	1.29 × 10^+01^	+	+	+	+
		Avg	1.73 × 10^+02^	1.60 × 10^+02^	2.83 × 10^+01^	1.13 × 10^+02^	1.71 × 10^+01^				
		SD	7.22 × 10^+00^	1.18 × 10^+01^	2.15 × 10^+01^	2.81 × 10^+01^	4.09 × 10^+00^				
*SF*_5_	150 k	Best	8.52 × 10^−14^	0.00 × 10^+00^	0.00 × 10^+00^	0.00 × 10^+00^	0.00 × 10^+00^	+	=	=	=
		Avg	4.03 × 10^−13^	0.00 × 10^+00^	0.00 × 10^+00^	0.00 × 10^+00^	0.00 × 10^+00^				
		SD	2.79 × 10^−13^	0.00 × 10^+00^	0.00 × 10^+00^	0.00 × 10^+00^	0.00 × 10^+00^				
*SF*_6_	150 k	Best	9.12 × 10^−08^	5.68 × 10^−14^	2.84 × 10^−14^	2.84 × 10^−14^	2.84 × 10^−14^	+	+	=	=
		Avg	1.01 × 10^−07^	9.09 × 10^−14^	2.84 × 10^−14^	2.84 × 10^−14^	2.84 × 10^−14^				
		SD	2.42 × 10^−08^	3.31 × 10^−14^	0.00 × 10^+00^	0.00 × 10^+00^	0.00 × 10^+00^				
*+/−/=*								21/0/0	20/0/1	18/0/3	17/2/2
*p-value*								0.000	0.000	0.000	0.001
*Significance (at 5%)*								Yes	Yes	Yes	Yes

‘+’, ‘−’ and ‘=’ represent the proposed scheme is significantly better, worse or equal, respectively, when compared with the competitor.

**Table 3:** Wilcoxon Rank Sum Test Results for SaMDE versus DE, SPIDE, DNDE and MRLDE.

Algorithms		ΣR^+^	ΣR^−^	z-Value	*p*-Value	Sig at α = 0.05
SaMDE vs.	DE	231	0	4.015	<0.001	+
	SPIDE	210	0	3.920	<0.001	+
	DNDE	171	0	3.724	<0.001	+
	MRLDE	182	8	3.501	<0.001	+

**Table 4:** Friedman’s Ranks and Critical difference (CD) calculated by Bonferroni–Dunn’s Method.

Rank	DE	SPIDE	DNDE	MRLDE	SaMDE	CD (α = 0.1)	CD (α = 0.05)
D = 30	4.95	3.50	2.90	2.40	1.24	1.0935	1.2189

**Table 5:** Comparison of SaMDE with SaDE, JADE, rJADE, APadapSS-JADE, SHADE and DEGOS in terms of average error and standard deviation (SD).

F	Max NFE	SaDE	JADE	rJADE	APadapSS-JADE	SHADE	DEGOS	SaMDE
*f*_1_	150k	4.6 × 10^−20 +^ (7.1 × 10^−20^)	1.9 × 10^−60 −^ (8.3 × 10^−60^)	1.8 × 10^−53 +^ (1.3 × 10^−52^)	2.4 × 10^−75 −^ (1.4 × 10^−74^)	1.1 × 10^−70 −^ (4.6 × 10^−70^)	3.6 × 10^−26 +^ (3.4 × 10^−26^)	4.7 × 10^−57^ (2.2 × 10^−57^)
*f*_2_	200K	2.0 × 10^−14 +^ (1.2 × 10^−14^)	1.9 × 10^−25 +^ (9.1 × 10^−25^)	1.6 × 10^−28 +^ (6.1 × 10^−28^)	1.8 × 10^−44^ (1.3 × 10^−43^)	4.7 × 10^−49 −^ (5.0 × 10^−49^)	4.5 × 10^−19 +^ (2.6 × 10^−19^)	6.3 × 10^−38^ (3.5 × 10^−38^)
*f*_3_	500k	9.3 × 10^−37 +^ (5.3 × 10^−36^)	6.0 × 10^−61 +^ (3.0 × 10^−60^)	1.6 × 10^+00^ (3.1 × 10^+00^)	2.50 × 10^−61 +^ (8.35 × 10^−61^)	5.5 × 10^−61 +^ (3.3 × 10^−61^)	1.7 × 10^−22 +^ (8.3 × 10^−22^)	5.6 × 10^−63^ (4.0 × 10^−63^)
*f*_4_	500k	7.2 × 10^−11 +^ (2.0 × 10^−10^)	8.1 × 10^−24 +^ (4.1 × 10^−23^)	1.1 × 10^−15 +^ (4.8 × 10^−16^)	5.14 × 10^−22 +^ (5.4 × 10^−22^)	2.1 × 10^−41 +^ (2.3 × 10^−41^)	4.4 × 10^−01 +^ (1.09 × 10^−01^)	4.3 × 10^−64^ (8.7 × 10^−64^)
*f*_5_	500k	2.0 × 10^+01 +^ (8.1 × 10^+00^)	8.1 × 10^−02 +^ (7.2 × 10^−01^)	2.2 × 10^−30 +^ (4.7 × 10^−30^)	3.1 × 10^−01 +^ (1.0 × 10^+00^)	7.9 × 10^−02 +^ (7.7 × 10^−02^)	3.2 × 10^−22 +^ (3.22 × 10^−22^)	2.1 × 10^−30^ (1.3 × 10^−30^)
*f*_6_	10k	9.1 × 10^+02 +^ (2.0 × 10^+02^)	2.9 × 10^+00 +^ (1.1 × 10^+00^)	1.2 × 10^+00 +^ (1.2 × 10^+00^)	1.0 × 10^+00 =^ (1.9 × 10^+00^)	2.6 × 10^+00 +^ (1.1 × 10^+00^)	5.8 × 10^+01 +^ (1.3 × 10^+02^)	1.1 × 10^+00^ (4.8 × 10^−01^)
*f*_7_	300k	5.0 × 10^−03 +^ (1.4 × 10^−03^)	6.6 × 10^−04 +^ (2.2 × 10^−04^)	4.8 × 10^−04 +^ (1.4 × 10^−04^)	5.9 × 10^−04 +^ (1.8 × 10^−04^)	5.9 × 10^−04 +^ (2.3 × 10^−04^)	2.1 × 10^−03 +^ (1.2 × 10^−04^)	3.0 × 10^−04^ (1.7 × 10^−04^)
*f*_8_	100k	4.8 × 10^+00 −^ (3.1 × 10^+01^)	3.0 × 10^−05 −^ (2.1 × 10^−05^)	4.2 × 10^−09 −^ (4.7 × 10^−09^)	1.8 × 10^−08 −^ (1.1 × 10^−07^)	1.5 × 10^−03 −^ (1.6 × 10^−03^)	2.1 × 10^+02 −^ (1.0 × 10^+02^)	6.3 × 10^+02^ (3.3 × 10^+02^)
*f*_9_	100k	1.6 × 10^−03 +^ (7.0 × 10^−04^)	1.1 × 10^−04 +^ (6.1 × 10^−05^)	1.2 × 10^−02 +^ (1.7 × 10^−02^)	2.9 × 10^−01 +^ (5.6 × 10^−01^)	1.7 × 10^−02 +^ (7.3 × 10^−02^)	1.5 × 10^+01 +^ (1.4 × 10^+01^)	1.0 × 10^−09^ (1.3 × 10^−09^)
*f*_10_	50k	2.9 × 10^−03 +^ (4.9 × 10^−04^)	8.9 × 10^−10 +^ (7.0 × 10^−10^)	3.5 × 10^−10 +^ (2.7 × 10^−10^)	4.1 × 10^−10 +^ (1.8 × 10^−11^)	2.6 × 10^−10 =^ (9.3 × 10^−10^)	5.4 × 10^−04 +^ (2.7 × 10^−04^)	2.7 × 10^−10^ (5.5 × 10^−09^)
*f*_11_	50k	7.9 × 10^−04 +^ (1.4 × 10^−03^)	9.4 × 10^−08 +^ (6.1 × 10^−07^)	1.1 × 10^−06 +^ (1.2 × 10^−06^)	0.0 × 10^+00 −^ (0.0 × 10^+00^)	1.6 × 10^−14 +^ (9.3 × 10^−14^)	3.1 × 10^−05 +^ (2.5 × 10^−05^)	2.9 × 10^−16^ (1.8 × 10^−16^)
*f*_12_	50k	2.0 × 10^−05 +^ (9.4 × 10^−06^)	4.4 × 10^−17 +^ (2.0 × 10^−16^)	1.8 × 10^−18 −^ (5.3 × 10^−18^)	2.2 × 10^−22 −^ (7.7 × 10^−22^)	3.6 × 10^−19 −^ (6.5 × 10^−19^)	7.8 × 10^−04 +^ (6.0 × 10^−04^)	2.0 × 10^−17^ (2.1 × 10^−17^)
*f*_13_	50k	6.2 × 10^−05 +^ (2.1 × 10^−05^)	2.1 × 10^−16 =^ (6.6 × 10^−16^)	1.5 × 10^−15 +^ (4.8 × 10^−15^)	3.7 × 10^−20 −^ (1.2 × 10^−19^)	3.8 × 10^−18 −^ (3.4 × 10^−18^)	5.9 × 10^−06 +^ (1.2 × 10^−06^)	1.2 × 10^−16^ (2.6 × 10^−16^)
SaMDE (*w/l/t*)		12/1/0 *p* = 0.003+	10/2/1 *p* = 0.022+	11/2/0 *p* = 0.022+	6/6/1 *p* = 1.00=	7/5/1 *p* = 0.774=	12/1/0 *p* = 0.003+	−

‘+’, ‘=’, ‘−’ means significant better, equal or worse, respectively, at α = 0.05.

**Table 6:** Result of Wilcoxon-rank sum test on the results obtained in Table 7.

Algorithms		Pairwise Rank	ΣR^+^	ΣR^−^	z Value	*p*-Value	Sig at α = 0.05
SaMDE *vs*	SaDE	(1.08, 1.92)	79	12	2.341	0.019	+
	JADE	(1.15, 1.85)	76	15	2.132	0.033	+
	rJADE	(1.15, 1.85)	74	17	1.992	0.046	+
	APAdapSS-JADE	(1.50, 1.50)	46	45	0.035	0.972	=
	SHADE	(1.46, 1.54)	48	30	0.706	0.480	=
	DEGOS	(1.08, 1.92)	78	13	2.271	0.023	+

‘+’ means significantly better and ‘=’ means significantly equal.

**Table 7:** Friedman Ranks Critical difference (CD) calculated by Bonferroni–Dunn’s procedure on the results obtained in Table 7.

	SaDE	JADE	rJADE	APadapSS-JADE	SHADE	DEGOS	SaMDE	CD (α = 0.1)	CD (α = 0.05)
Rank	6.17	4.00	3.75	2.71	2.96	6.00	2.42	2.02	2.23

**Table 8:** Performance evaluation of SaMDE on IEEE CEC2017 for *D* = 30 in terms of average error and standard deviation.

***F***	***SaMDE***		***TRADE***		***DEexp***		***iLSHADE***		**PAIDDE**	
	**Mean**	**SD**	**Mean**	**SD**	**Mean**	**SD**	**Mean**	**SD**	**Mean**	**SD**
***C*_1_**	0.00 × 10^+00^	0.00 × 10^+00^	0.00 × 10^+00^	0.00 × 10^+00^	0.00 × 10^+00^	0.00 × 10^+00^	0.00 × 10^+00^	0.00 × 10^+00^	0.00 × 10^+00^	0.00 × 10^+00^
***C*_3_**	0.00 × 10^+00^	0.00 × 10^+00^	2.31 × 10^+01^	4.16 × 10^+01^	0.00 × 10^+00^	0.00 × 10^+00^	5.57 × 10^−15^	1.71 × 10^−14^	0.00 × 10^+00^	0.00 × 10^+00^
***C*_4_**	5.85 × 10^+01^	0.00 × 10^+00^	5.97 × 10^+01^	2.34 × 10^+00^	5.90 × 10^+01^	1.51 × 10^+00^	5.77 × 10^+01^	8.41 × 10^+00^	5.85 × 10^+01^	1.15 × 10^−14^
***C*_5_**	1.14 × 10^+01^	3.85 × 10^+00^	1.91 × 10^+01^	4.64 × 10^+00^	9.83 × 10^+00^	2.75 × 10^+00^	7.76 × 10^+00^	1.70 × 10^+00^	6.85 × 10^+00^	1.48 × 10^+00^
***C*_6_**	1.13 × 10^−11^	6.12 × 10^−08^	0.00 × 10^+00^	0.00 × 10^+00^	2.60 × 10^−09^	1.71 × 10^−08^	1.21 × 10^−08^	5.04 × 10^−08^	4.56 × 10^−09^	2.49 × 10^−08^
***C*_7_**	4.12 × 10^+01^	4.23 × 10^+00^	5.47 × 10^+01^	9.38 × 10^+00^	4.19 × 10^+01^	3.20 × 10^+00^	3.79 × 10^+01^	1.47 × 10^+00^	3.72 × 10^+01^	1.33 × 10^+00^
***C*_8_**	1.04 × 10^+00^	8.54 × 10^+00^	2.01 × 10^+01^	4.24 × 10^+00^	1.06 × 10^+01^	3.12 × 10^+01^	7.45 × 10^+00^	1.78 × 10^+00^	7.10 × 10^+00^	1.12 × 10^+00^
***C*_9_**	0.00 × 10^+00^	0.00 × 10^+00^	0.00 × 10^+00^	0.00 × 10^+00^	0.00 × 10^+00^	0.00 × 10^+00^	0.00 × 10^+00^	0.00 × 10^+00^	0.00 × 10^+00^	0.00 × 10^+00^
***C*_10_**	1.06 × 10^+03^	2.25 × 10^+02^	7.29 × 10^+03^	3.11 × 10^+02^	1.42 × 10^+03^	2.15 × 10^+02^	1.74 × 10^+03^	3.15 × 10^+02^	1.41 × 10^+03^	2.53 × 10^+02^
***C*_11_**	4.97 × 10^+00^	2.86 × 10^+00^	1.29 × 10^+01^	1.91 × 10^+01^	3.63 × 10^+00^	4.40 × 10^+00^	1.42 × 10^+01^	2.25 × 10^+01^	1.91 × 10^+01^	2.54 × 10^+01^
***C*_12_**	1.19 × 10^+03^	5.54 × 10^+03^	1.30 × 10^+04^	8.83 × 10^+03^	3.87 × 10^+02^	2.02 × 10^+02^	8.80 × 10^+02^	3.91 × 10^+02^	1.05 × 10^+03^	3.91 × 10^+02^
***C*_13_**	1.79 × 10^+01^	1.63 × 10^+01^	2.45 × 10^+01^	5.61 × 10^+00^	1.46 × 10^+01^	6.90 × 10^+00^	1.85 × 10^+01^	8.47 × 10^+00^	1.63 × 10^+01^	6.14 × 10^+00^
***C*_14_**	1.19 × 10^+01^	6.81 × 10^+00^	2.41 × 10^+01^	5.81 × 10^+00^	1.47 × 10^+01^	7.49 × 10^+00^	2.18 × 10^+01^	1.08 × 10^+00^	2.07 × 10^+01^	4.95 × 10^+00^
***C*_15_**	3.32 × 10^+00^	1.72 × 10^+00^	6.65 × 10^+00^	2.59 × 10^+00^	3.41 × 10^+00^	2.00 × 10^+00^	3.70 × 10^+00^	1.94 × 10^+00^	3.36 × 10^+00^	1.70 × 10^+01^
***C*_16_**	2.69 × 10^+01^	1.17 × 10^+01^	1.56 × 10^+01^	9.27 × 10^+00^	8.41 × 10^+01^	8.64 × 10^+01^	4.88 × 10^+01^	6.91 × 10^+01^	6.58 × 10^+01^	8.32 × 10^+01^
***C*_17_**	2.42 × 10^+01^	4.55 × 10^+00^	2.72 × 10^+01^	2.76 × 10^+00^	2.66 × 10^+01^	8.90 × 10^+00^	3.81 × 10^+01^	5.23 × 10^+00^	3.34 × 10^+01^	6.61 × 10^+00^
***C*_18_**	2.76 × 10^+01^	2.56 × 10^+00^	2.59 × 10^+01^	1.02 × 10^+01^	2.10 × 10^+01^	1.69 × 10^+00^	2.14 × 10^+01^	8.15 × 10^−01^	2.21 × 10^+01^	1.28 × 10^+00^
***C*_19_**	5.12 × 10^+00^	3.46 × 10^+00^	5.54 × 10^+00^	1.69 × 10^+00^	5.22 × 10^+00^	1.40 × 10^+00^	8.53 × 10^+00^	1.97 × 10^+00^	6.52 × 10^+00^	1.89 × 10^+00^
***C*_20_**	1.19 × 10^+01^	1.36 × 10^+00^	2.07 × 10^+01^	6.92 × 10^+00^	2.88 × 10^+00^	3.09 × 10^+01^	4.80 × 10^+01^	1.86 × 10^+01^	3.32 × 10^+01^	6.44 × 10^+00^
***C*_21_**	2.22 × 10^+02^	6.14 × 10^+00^	2.20 × 10^+02^	4.90 × 10^+00^	2.10 × 10^+02^	3.06 × 10^+00^	2.08 × 10^+01^	1.65 × 10^+00^	2.07 × 10^+02^	1.48 × 10^+00^
***C*_22_**	1.00 × 10^+02^	0.00 × 10^+00^	1.00 × 10^+02^	0.00 × 10^+00^	1.00 × 10^+02^	1.01 × 10^−01^	1.00 × 10^+02^	1.00 × 10^−13^	1.00 × 10^+02^	0.00 × 10^+00^
***C*_23_**	3.58 × 10^+02^	7.79 × 10^+00^	3.62 × 10^+02^	8.58 × 10^+00^	3.45 × 10^+02^	4.40 × 10^+00^	3.51 × 10^+02^	4.50 × 10^+00^	3.48 × 10^+02^	2.53 × 10^+00^
***C*_24_**	2.34 × 10^+02^	6.41 × 10^+00^	4.42 × 10^+02^	5.44 × 10^+00^	4.22 × 10^+02^	3.12 × 10^+00^	4.25 × 10^+02^	2.70 × 10^+00^	4.25 × 10^+02^	1.38 × 10^+00^
***C*_25_**	3.86 × 10^+02^	6.33 × 10^−02^	3.87 × 10^+02^	2.69 × 10^−02^	3.86 × 10^+02^	1.29 × 10^−02^	3.87 × 10^+02^	2.40 × 10^−02^	3.86 × 10^+02^	2.73 × 10^−02^
***C*_26_**	1.15 × 10^+03^	2.45 × 10^+02^	9.88 × 10^+02^	8.03 × 10^+01^	8.59 × 10^+02^	4.77 × 10^+01^	9.08 × 10^+02^	4.67 × 10^+01^	9.23 × 10^+02^	3.32 × 10^+01^
***C*_27_**	4.98 × 10^+02^	2.51 × 10^+00^	4.93 × 10^+02^	1.15 × 10^+01^	5.00 × 10^+02^	8.00 × 10^+00^	5.04 × 10^+02^	6.86 × 10^+00^	5.02 × 10^+02^	5.10 × 10^+00^
***C*_28_**	3.01 × 10^+02^	5.63 × 10^+01^	3.32 × 10^+02^	5.17 × 10^+01^	3.27 × 10^+02^	3.19 × 10^+02^	4.77 × 10^+01^	4.12 × 10^+01^	3.22 × 10^+02^	4.63 × 10^+01^
***C*_29_**	4.58 × 10^+02^	1.26 × 10^+02^	4.05 × 10^+02^	2.63 × 10^+01^	4.26 × 10^+02^	1.61 × 10^+01^	4.46 × 10^+02^	1.05 × 10^+01^	4.34 × 10^+02^	8.34 × 10^+00^
***C*_30_**	1.95 × 10^+03^	8.40 × 10^+01^	2.06 × 10^+03^	5.17 × 10^+01^	2.00 × 10^+03^	3.73 × 10^+01^	2.03 × 10^+03^	5.90 × 10^+01^	1.99 × 10^+03^	8.32 × 10^+01^
***w/l/t***			19/7/3		15/9/5		17/9/3		14/9/6	
***F***	***DEGoS***		***CJADE***		***IMODE***		***SHADE***		***HMRFO***	
	**Mean**	**SD**	**Mean**	**SD**	**Mean**	**SD**	**Mean**	**SD**	**Mean**	**SD**
***C*_1_**	0.00 × 10^+00^	0.00 × 10^+00^	0.00 × 10^+00^	0.00 × 10^+00^	9.11 × 10^−11^	1.50 × 10^−03^	0.00 × 10^+00^	0.00 × 10^+00^	1.01 × 10^−11^	2.46 × 10^+03^
***C*_3_**	1.82 × 10^−01^	9.97 × 10^−01^	1.22 × 10^−04^	1.80 × 10^+04^	1.91 × 10^−07^	8.30 × 10^−09^	0.00 × 10^+00^	0.00 × 10^+00^	2.70 × 10^+02^	6.90 × 10^+05^
***C*_4_**	4.98 × 10^+01^	2.23 × 10^+02^	4.78 × 10^+01^	2.37 × 10^+02^	2.21 × 10^+01^	2.87 × 10^+02^	4.09 × 10^+01^	2.75 × 10^+02^	4.76 × 10^+01^	3.61 × 10^+01^
***C*_5_**	3.60 × 10^+01^	3.65 × 10^+02^	2.67 × 10^+01^	4.80 × 10^+00^	2.63 × 10^+02^	4.17 × 10^+00^	1.79 × 10^+01^	2.30 × 10^+00^	4.49 × 10^+01^	3.63 × 10^+01^
***C*_6_**	5.52 × 10^−06^	1.85 × 10^−05^	0.00 × 10^+00^	0.00 × 10^+00^	5.82 × 10^+01^	6.39 × 10^+00^	0.00 × 10^+00^	0.00 × 10^+00^	6.47 × 10^+00^	2.07 × 10^+01^
***C*_7_**	1.64 × 10^+02^	5.55 × 10^+01^	5.46 × 10^+01^	3.41 × 10^+00^	9.22 × 10^+02^	3.14 × 10^+02^	4.83 × 10^+01^	3.09 × 10^+00^	1.56 × 10^+02^	1.01 × 10^+00^
***C*_8_**	7.68 × 10^+01^	7.25 × 10^+01^	2.71 × 10^+01^	4.64 × 10^+00^	2.19 × 10^+01^	4.00 × 10^+01^	1.86 × 10^+01^	3.04 × 10^+00^	2.31 × 10^+01^	1.78 × 10^+01^
***C*_9_**	7.57 × 10^−02^	3.23 × 10^−01^	5.96 × 10^−03^	2.27 × 10^−03^	5.59 × 10^+03^	1.52 × 10^+03^	0.00 × 10^+00^	0.00 × 10^+00^	6.21 × 10^+02^	4.86 × 10^+01^
***C*_10_**	5.41 × 10^+03^	2.26 × 10^+03^	1.91 × 10^+03^	2.34 × 10^+01^	3.81 × 10^+03^	4.76 × 10^+02^	1.94 × 10^+03^	2.18 × 10^+02^	2.35 × 10^+03^	6.44 × 10^+02^
***C*_11_**	1.73 × 10^+01^	2.05 × 10^+01^	2.84 × 10^+01^	2.05 × 10^+01^	1.96 × 10^+02^	4.83 × 10^+01^	2.68 × 10^+01^	2.70 × 10^+01^	3.71 × 10^+01^	1.21 × 10^+01^
***C*_12_**	8.59 × 10^+03^	8.50 × 10^+03^	1.26 × 10^+03^	7.07 × 10^+02^	1.12 × 10^+03^	3.75 × 10^+02^	1.76 × 10^+03^	1.34 × 10^+03^	1.87 × 10^+03^	1.31 × 10^+04^
***C*_13_**	3.32 × 10^+01^	2.37 × 10^+01^	5.53 × 10^+01^	3.04 × 10^+01^	3.98 × 10^+02^	1.70 × 10^+02^	3.83 × 10^+01^	2.10 × 10^+01^	7.08 × 10^+01^	1.06 × 10^+04^
***C*_14_**	2.49 × 10^+01^	7.38 × 10^+00^	4.69 × 10^+03^	1.26 × 10^+04^	1.92 × 10^+02^	5.61 × 10^+01^	2.86 × 10^+01^	9.02 × 10^+00^	5.62 × 10^+02^	9.00 × 10^+02^
***C*_15_**	9.62 × 10^+00^	5.33 × 10^+00^	3.22 × 10^+01^	2.32 × 10^+01^	2.15 × 10^+02^	8.73 × 10^+01^	1.64 × 10^+01^	1.36 × 10^+01^	3.58 × 10^+01^	3.60 × 10^+03^
***C*_16_**	1.62 × 10^+02^	2.99 × 10^+02^	4.19 × 10^+02^	1.59 × 10^+02^	1.48 × 10^+03^	4.69 × 10^+02^	3.32 × 10^+02^	1.28 × 10^+02^	3.34 × 10^+02^	2.76 × 10^+02^
***C*_17_**	3.82 × 10^+01^	1.40 × 10^+01^	6.88 × 10^+01^	1.77 × 10^+01^	8.73 × 10^+02^	2.64 × 10^+02^	4.52 × 10^+01^	1.10 × 10^+01^	1.36 × 10^+02^	1.35 × 10^+02^
***C*_18_**	4.33 × 10^+01^	5.51 × 10^+01^	5.68 × 10^+03^	2.14 × 10^+04^	1.61 × 10^+02^	7.49 × 10^+01^	6.65 × 10^+01^	5.64 × 10^+01^	6.81 × 10^+02^	4.46 × 10^+04^
***C*_19_**	6.06 × 10^+00^	2.51 × 10^+00^	6.30 × 10^+02^	2.43 × 10^+03^	5.96 × 10^+02^	3.58 × 10^+02^	8.67 × 10^+00^	3.31 × 10^+00^	1.43 × 10^+02^	2.51 × 10^+03^
***C*_20_**	3.31 × 10^+01^	3.92 × 10^+01^	1.16 × 10^+02^	5.88 × 10^+01^	6.78 × 10^+02^	1.95 × 10^+02^	9.65 × 10^+01^	5.34 × 10^+01^	1.27 × 10^+02^	1.24 × 10^+02^
***C*_21_**	2.39 × 10^+02^	4.81 × 10^+01^	2.25 × 10^+02^	4.20 × 10^+00^	4.16 × 10^+02^	3.21 × 10^+01^	2.19 × 10^+02^	3.76 × 10^+00^	2.40 × 10^+02^	1.79 × 10^+01^
***C*_22_**	1.00 × 10^+02^	8.30 × 10^−14^	1.00 × 10^+02^	5.60 × 10^−05^	1.33 × 10^+03^	1.96 × 10^+03^	1.00 × 10^+02^	0.00 × 10^+00^	2.37 × 10^+02^	3.07 × 10^−13^
***C*_23_**	3.75 × 10^+02^	2.89 × 10^+01^	3.72 × 10^+02^	4.78 × 10^+00^	7.98 × 10^+02^	8.40 × 10^+01^	3.65 × 10^+02^	4.86 × 10^+00^	4.08 × 10^+02^	2.37 × 10^+01^
***C*_24_**	4.49 × 10^+02^	2.81 × 10^+01^	4.40 × 10^+02^	3.32 × 10^+00^	9.59 × 10^+02^	7.34 × 10^+01^	4.36 × 10^+02^	3.52 × 10^+00^	4.91 × 10^+02^	2.04 × 10^+01^
***C*_25_**	3.86 × 10^+02^	6.34 × 10^−01^	3.87 × 10^+02^	1.41 × 10^−01^	3.94 × 10^+02^	1.82 × 10^+01^	3.87 × 10^+02^	1.79 × 10^−01^	3.87 × 10^+02^	1.30 × 10^+01^
***C*_26_**	1.20 × 10^+03^	2.45 × 10^+02^	1.17 × 10^+03^	7.74 × 10^+01^	4.43 × 10^+03^	1.14 × 10^+03^	1.10 × 10^+03^	6.07 × 10^+01^	1.41 × 10^+03^	8.08 × 10^+02^
***C*_27_**	5.00 × 10^+02^	1.14 × 10^+01^	5.03 × 10^+02^	6.70 × 10^+00^	7.60 × 10^+02^	1.25 × 10^+02^	5.03 × 10^+02^	6.50 × 10^+00^	5.31 × 10^+02^	1.52 × 10^+01^
***C*_28_**	3.44 × 10^+02^	5.71 × 10^+01^	3.28 × 10^+02^	4.77 × 10^+01^	3.36 × 10^+02^	5.80 × 10^+01^	3.39 × 10^+02^	5.31 × 10^+01^	3.32 × 10^+02^	5.51 × 10^+01^
***C*_29_**	4.13 × 10^+02^	3.82 × 10^+01^	4.71 × 10^+02^	3.57 × 10^+01^	1.56 × 10^+03^	4.14 × 10^+02^	4.65 × 10^+02^	1.90 × 10^+01^	5.70 × 10^+02^	1.68 × 10^+02^
***C*_30_**	2.13 × 10^+03^	1.21 × 10^+02^	2.15 × 10^+03^	1.66 × 10^+02^	4.36 × 10^+03^	1.42 × 10^+03^	2.08 × 10^+03^	1.08 × 10^+02^	2.32 × 10^+03^	1.09 × 10^+03^
***w/l/t***	24/2/3		25/2/2		27/2/0		21/4/4		27/2/0	

**Table 9:** Result of Wilcoxon-rank sum test on the results obtained in Table 8.

Algorithms		Pairwise Rank	ΣR^+^	ΣR^−^	z Value	*p*-Value	Sig at α = 0.05
SaMDE *vs.*	TRADE	(1.29, 1.71)	271	080	2.426	0.015	+
	DE*exp*	(1.40, 1.60)	168	132	0.514	0.607	=
	iLSHADE	(1.34, 1.66)	211	114	1.305	0.192	=
	PAIDDE	(1.41, 1.59)	170	105	0.989	0.323	=
	DEGoS	(1.12, 1.88)	326	025	3.821	<0.001	+
	CJADE	(1.11, 1.89)	344	007	4.280	<0.001	+
	IMODE	(1.07, 1.93)	421	014	4.400	<0.001	+
	SHADE	(1.21, 1.79)	289	036	3.404	<0.001	+
	HMRFO	(1.07, 1.93)	421	014	4.400	<0.001	+

‘+’ means significantly better and ‘=’ means significantly equal.

**Table 10:** Friedman Ranks Critical difference (CD) calculated by Bonferroni–Dunn’s procedure on the results obtained in Table 8.

	SaMDE	TRADE	DEexp	iLSHADE	PAIDDE	DEGoS	CJADE	IMODE	SHADE	HMRFO	CD (α = 0.1)	CD (α = 0.05)
Rank	3.00	4.89	3.05	4.02	3.36	6.46	6.98	9.04	5.82	8.38	2.018	2.204

**Table 11:** Experimental results for molecular potential energy problem in terms of fitness value.

*No. of Beads*	Max− NFE	Fitness	DE	TDE	DERL	MRLDE	SaMDE
*n* = 10	50,000	Best	−0.589389	−0.589389	−0.589389	−0.589389	−0.589389
		Worst	−0.507152	−0.507152	−0.576621	−0.589338	−0.589389
		Mean	−0.523599	−0.556494	−0.581728	−0.589368	−0.589389
		SD	3.28 × 10^−02^	4.02 × 10^−02^	6.25 × 10^−03^	2.47 × 10^−05^	0.00 × 10^−00^
*n* = 15	150,000	Best	−0.493301	−0.493409	−0.493420	−0.493420	−0.493420
		Worst	−0.328946	−0.258712	−0.247861	−0.347986	−0.411183
		Mean	−0.263733	−0.296743	−0.329177	−0.365739	−0.476932
		SD	1.08 × 10^−01^	6.55 × 10^−02^	5.76 × 10^−02^	5.57 × 10^−02^	3.28 × 10^−02^
*n* = 20	200,000	Best	−0.673352	−0.995013	−1.009910	−1.000530	−1.000570
		Worst	−0.344406	−0.508879	−0.426067	−0.673352	−0.836098
		Mean	−0.606527	−0.623435	−0.705211	−0.853121	−0.918450
		SD	1.31 × 10^−01^	8.36 × 10^−02^	1.52 × 10^−01^	8.33 × 10^−02^	7.35 × 10^−02^
*n* = 25	250,000	Best	−0.830098	−0.904371	−0.904489	−0.904500	−0.904501
		Worst	−0.165625	−0.247286	−0.395887	−0.330674	−0.494571
		Mean	−0.248202	−0.362993	−0.412335	−0.462137	−0.576693
		SD	1.03 × 10^−01^	8.38 × 10^−02^	1.59 × 10^−01^	1.33 × 10^−01^	1.66 × 10^−01^

## Data Availability

All related data is contained within the article.
